# Economic Evaluation of Add-on Levetiracetam for the Treatment of Refractory Partial Epilepsy in Korea

**DOI:** 10.4306/pi.2009.6.3.185

**Published:** 2009-06-23

**Authors:** Guk-Hee Suh, Sang Keol Lee

**Affiliations:** Department of Psychiatry, Hallym University Medical Center, Hangang Sacred Heart Hospital, Seoul, Korea.

**Keywords:** Levetiracetam, Epilepsy, Partial seizures, Seizure-free day, Quality adjusted life year, Cost

## Abstract

**Objective:**

This study estimated the expected cost-effectiveness ratio expressed as the incremental cost per seizure-free day (SFD) gained and the incremental cost per quality adjusted life year (QALY) gained when using levetiracetam (LEV) as add-on therapy from a third-party payer perspective.

**Methods:**

A 1-year dose-escalation decision-tree model comparing LEV plus standard therapy (ST) with ST alone was designed to combine transition probabilities, costs and outcomes. The short-term outcomes and probabilities were derived from a prospective, open-label clinical trial with 100 Korean adults with refractory partial epilepsy. All data for the direct medical costs were derived from Korean cost data extracted from reports published by the National Health Insurance Corporation.

**Results:**

The average gain in SFDs attributed to LEV add-on was 18.3 days per patient per year and the incremental cost-effectiveness ratios (ICERs) for LEV add-on were US$ 44 per SFD per patient and US$ 11,084 per QALY gained. All sensitivity analyses showed that the model was robust to the assumptions made.

**Conclusion:**

The economic evaluation indicates that, given a wide range of assumptions, the increased cost of treating patients having refractory partial epilepsy with LEV may be partially offset by a reduction in other direct medical costs. This reduction is a consequence of an increase in the number of SFDs and improved quality of life.

## Introduction

Epilepsy is a common neurological condition, with an estimated incidence of 50 per 100,000 and prevalence of 5-10 per 1,000 in the developed world.^1^ The majority of the population with a diagnosis of epilepsy will go into remission when treated with an appropriate antiepileptic drug (AED). However, up to 30% will develop refractory epilepsy.^2^ The majority of patients with refractory epilepsy have localization-related (partial) seizures, which are divided into three types: simple partial; complex partial and secondarily generalized tonic-clonic seizures.^3^

Over the past two decades there has been a rapid expansion in the number of novel AEDs. One such drug is levetiracetam (LEV). LEV is a newer AED that has been approved as an add-on therapy for refractory partial epilepsy.^4^-^6^ LEV has almost ideal pharmacokinetic characteristics that include high oral bioavailability, linear pharmacokinetics, low plasma protein binding, primary excretion unchanged in urine, and no known clinically significant drug-drug interactions, with proven efficacy and tolerability.^7^,^8^ Studies have been conducted in several countries including ethnically diverse populations and confirmed that ethnicity does not have any impact on the pharmacokinetic parameters of LEV.^9^,^10^

About 95 percent of the Korean population receive health care through national health insurance, and the remaining 5 percent who are unable to pay receive insurance coverage from the government. Health care costs are rapidly rising due to characteristics of the health care delivery system and other factors (i.e., population aging, innovative technologies, and higher patient expectation following increasing health consciousness).

In addition to presenting medical, psychological and social burdens, epilepsy also constitutes an economic problem for society. The economic consequences of epilepsy are manifested both in the direct costs for the treatment of the disease and in the indirect costs, which include loss of earnings due to reduced productivity.^11^ A better understanding of the economic aspects of epilepsy will assist decision makers in identifying the most cost-effective treatment in order to make the best use of limited available health resources.

This study aimed to estimate the expected cost-effectiveness ratio expressed as the incremental cost per seizure-free day (SFD) gained and the incremental cost per quality adjusted life year (QALY) gained when using LEV as add-on therapy compared to standard therapy (ST) alone.

## Methods

Economic modelling was used to extrapolate cost and outcome estimates over a longer time period than that which can be obtained from clinical trial data. This study was performed through the design of a decision-tree model using TreeAge Pro 2006 Suite® Release 0.2, a health economic tool for conducting economic evaluations (http://www.treeage.com). This economic evaluation was performed from the third-party payer perspective (i.e., the Korean National Health Insurance Corporation).

Due to the 1-year time horizon of this economic evaluation, the cost and effectiveness measures were not discounted.

### Setting and data collection

For the economic evaluation the raw data from a detailed, open-label, single-arm clinical trial on the efficacy and safety of LEV in Korea was used.^12^ The study population consisted of 100 adult patients aged 18 years or older who received LEV as add-on therapy in addition to conventional AEDs. All the patients came from nine research centres in Korea and had uncontrolled epilepsy and/or intolerable adverse events on conventional AEDs.

Data from the 16-week clinical trial were recorded prospectively. It consisted of two periods: 1) an up-titration period (first 4 weeks) and 2) a maintenance period (for the following 12 weeks). For purposes of our analysis, data were extracted from the collected dataset for the period of 12 weeks before and 16 weeks after the first day of add-on therapy of LEV. Recorded data covered the following domains:

- Sociodemographics: age, gender, education, marital status, etc
- Type of epilepsy
- Seizure frequency for the period of 12 weeks before and 16 weeks after the first day of add-on therapy of LEV
- Retention rate of LEV
- Quality of life (QoL): 31-item Quality of Life in Epilepsy Inventory (QOLIE-31)^13^

When estimates of input parameters were not available, endorsed comments from the Scientific Advisory Board (SAB), convened by the Korean Epilepsy Society, were considered.

### Epilepsy-related quality of life assessment

Epilepsy-related QoL was assessed twice using the Korean translation of the QOLIE-31 at baseline before the beginning of add-on therapy of LEV (Week 0) and at the end of the clinical trial (Week 16), or earlier if withdrawn. The QOLIE-31 is a self-administered questionnaire designed to be completed by patients.^13^ Validation studies of the QOLIE-31 in different language versions also demonstrated good reliabilities and validities.^14^-^16^ QOLIE-31 was derived from the longer QOLIE-89, an instrument with 17 subscales, including generic and epilepsy-specific issues, and a separate item on health status (not scored).^17^ The QOLIE-31 includes 7 subscales: seizure worry, overall QoL, emotional wellbeing, energy-fatigue, cognitive functioning, medication effects, social function, and health status (not scored). The subscale and the total scores are calculated according to the algorithm defined by the author with scores ranging from 0 to 100 and higher scores indicating better function.^13^

### Study model

**FIGURE 1:**
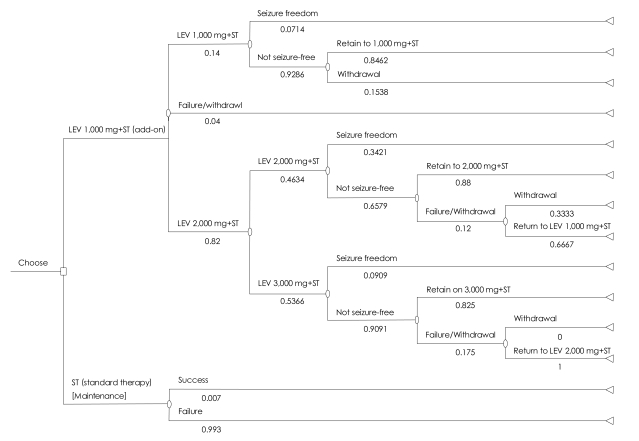
Decision tree for the management of treatment of refractory epileptic patients with combination therapy [Levetiracetam (LEV)+standard therapy] versus standard therapy alone.

A 1-year dose-escalation decision-tree model was designed for economic evaluation of LEV as an add-on therapy and further, was adapted to Korean data.^18^,^19^ Two hypothetical cohorts of patients were started either on combination therapy (LEV 1,000 mg/day plus ST) or on ST alone. For patients in the LEV arm the dose was increased up to 3,000 mg/day if freedom from seizures was not attained at lower doses (Figure 1). Patients who started on ST alone were kept in that arm for the whole 1-year duration of the economic model. In this model, success was defined as freedom from seizure, while failure was defined as withdrawal leading to being returned to ST in the combination therapy arm or not-seizure-free, continued refractory partial epilepsy state in the ST alone arm. All these assumptions were reviewed and endorsed by the SAB.

- Patients stayed on a specific LEV dose for 0.5 months before the dose could be maintained, changed, or stopped.
- Patients who experienced intolerable adverse events under higher doses of LEV could return to lower doses of LEV if they had experienced a relatively significant reduction in the frequency of seizures while treated with lower doses (i.e., 50% reduction in seizure frequency).
- Patients who stopped LEV returned to ST alone for the remaining period.
- Costs and outcomes of patients who returned to ST alone during LEV treatment were the same as those of patients who start with the ST alone.
- Costs and outcomes of patients who started on ST alone remained unchanged for the whole 1-year duration of the economic model.

More specifically, patients in the combination therapy arm started with LEV 1,000 mg/day plus ST for a 0.5-month observation period, after which they entered into one of the following pathways:

1. Stayed at LEV 1,000 mg/day plus ST for the remained 11.5 months if they attained complete seizure freedom.
2. Stopped LEV and return to ST alone for 11.5 months if they could not tolerate the LEV 1,000 mg/day plus ST.
3. Increased to LEV 2,000 mg/day if not seizure-free at the LEV 1,000 mg/day plus ST, but the LEV 1,000 mg/day dosage was tolerated.

Patients increased to LEV 2,000 mg/day and maintained that dosage for a 0.5-month observation period after which they entered one of the following pathways:

1. Stayed at LEV 2,000 mg/day plus ST for the remaining 11 months if they attained complete freedom from seizures.
2. Returned to the LEV 1,000 mg/day plus ST and maintain for 11 months if they could not tolerate the daily dose of LEV 2,000 mg but experienced significant reduction in the frequency of seizures at the LEV 1,000 mg/day plus ST.
3. Stopped LEV and return to ST alone for 11 months if they could not tolerate LEV 2,000 mg/ day and if they did not experience sufficient reduction in the frequency of seizures while treated with the 1,000 mg/day plus ST.
4. Increased dosage to LEV 3,000 mg/day if not seizure-free at the LEV 2,000 mg/day plus ST, but the 2,000 mg/day dosage was tolerated.

Patients increased to LEV 3,000 mg/day and maintain for a 0.5-month observation period after which they entered one of the following pathways:

1. Stayed at LEV 3,000 mg/day and ST for the remained 10.5 months if they attained complete freedom from or attained a significant reduction in the frequency of seizures.
2. Returnedto LEV 2,000 mg/day and maintained for 10.5 months if they did not tolerate the daily dose of 3,000 mg but experienced significant reduction in the frequency of seizures while treated with LEV 2,000 mg/day.
3. Stopped LEV and returned to ST alone for 10.5 months if they did not experience sufficient reduction in the frequency of seizures while treated with any dose of LEV add-on therapy.

The probabilities of each state for the combination therapy (LEV+ST) were derived from the outcomes of the 16-week duration of the aforementioned, open-label clinical trial.^12^ The transition probabilities for ST were adopted from a previous report.^18^

### Cost estimates

The economic evaluation focused on the direct medical costs incurred by the patients from the third-party payer perspective. LEV has been available since January 2007. Costs for ST should not include cost for LEV. Therefore, unit costs were obtained from the "2006 Korean National Health Insurance Statistical Year-book" and the "2006 Major Health Insurance Statistics".^20^,^21^ The average currency exchange rate in the year of 2006 was 955.51 Korea Won per US$ 1 (http://www.keb.co.kr/IBS/welcome.jsp). Resource use related to the routine care provided to patients with refractory partial epilepsy could not be derived from the aforementioned prospective clinical trial because information on healthcare resource utilization had not been collected.^12^ Furthermore, specific data on health resource utilization of patients with refractory epilepsy were not available in Korea. Therefore, routine follow-up of patients with epilepsy in typical practices was based on national data sources (gross costing method).^20^ It was assumed that patients with refractory epilepsy would incur higher costs as a consequence of frequent outpatient visits and hospital admissions. Success in the treatment of patients with refractory epilepsy would save greater amounts of money than success in all patients with epilepsy. It was also expected that use of the costs of all patients with epilepsy could underestimate potential economic benefits in this study. It is also a more conservative estimate than the usual cost measure. To cover medical costs for patients with refractory partial epilepsy, costs per patient for inpatient, outpatient, and pharmacy for a 1-year period were considered in this study. Costs consist of: inpatient (US$ 1,841 per person per year); outpatient (US$ 183 per person per year); and pharmacy (US$ 368 per person per year) for ST (Table 1); while for combination therapy, costs for LEV (US$ 0.97, US$ 1.45, and US$ 2.18 per tablet with LEV 250 mg, 500 mg, and 1,000 mg respectively)(http://www.kpis.or.kr/index.jsp) were added to the costs for s ST alone.

Generally speaking, reduced costs are assumed when seizures occur less frequently. The SAB endorsed an assumption of a linear relation between costs for inpatient and seizure frequency among patients with refractory partial epilepsy. For the base case scenario, in patients with seizure freedom (seizure frequency=0), the inpatient cost was considered to be zero. The better response was assumed for those patients who stayed at a certain dose of LEV per the clinician's judgement that the dose was sufficiently effective and tolerable for the patient. P^better response^=0.5 means that those with a better response may only need half the given inpatient cost. On the other hand, worse response was assumed for those patients who returned to a lower dose of LEV per the clinician's judgement that the patient was unable to tolerate a higher dose, but experienced a significant reduction in the frequency of seizures while treated with the lower dose of LEV. P^worse response^=0.8 means that those patients with worse response may need 80% of the inpatient cost.

### Effectiveness estimates

#### Seizure-Free Days Gained

Effectiveness was measured in terms of SFDs gained per year; SFDs are reported days without seizure. The metric 'SFDs gained' tries to address the patient's perspective by accounting for an improved ability to perform daily tasks on a day without seizure.^22^ It is also a more conservative measure than the usual efficacy measure based on the reduction of seizure frequency. The impact of adding LEV to the number of SFDs per year in patients with refractory partial epilepsy was assessed. Effectiveness estimates used in the CEA are derived from raw data from clinical trials in Korea^12^ and presented in Table 2.

#### Quality Adjusted Life Years Gained

The disease-specific health-status instrument, QOLIE-31, measures a range of health attributes from its 7 subscales including 1) seizure worry, 2) overall QoL, 3) emotional wellbeing, 4) energy-fatigue, 5) cognitive functioning, 6) medication effects, and 7) social function, as well as general health status (not scored). Subscale scores range from 0 to 100. In their validation paper, the authors who developed the QOLIE reported utility weights for above 7 dimensions by using econometric techniques (i.e., ordinary least squares regression).^13^ Utility is a measure of the preference for, or desirability of, a specific level of health state or specific health outcome (on a scale of 0 to 1). QoL is measured in utility weights, which are used to represent preferences for health states. In this study, the utility scores of each patient were calculated as follows:

Utility of a patient=Σ(subscale score i/100)×(weight i/sum of weights)

The mean value of the utilities of patients at each terminal node in the decision-tree model (Figure 1) was assigned as a utility, expressed as QALYs gained, at each terminal node.

### Incremental cost-effectiveness ratio

Measurement of the incremental cost-effectiveness ratios (ICER), i.e. incremental cost per SFD and the QALY gained, was derived from the trial in Korea. ICER was calculated as follows^*^:

ICER=(C_LEV+ST_-C_ST_)/(E_LEV+ST_-E_ST_)=ΔC/ΔE

^*^C: cost, E: effectiveness, Δ: difference

The net cost is equal to the total cost of combination therapy less the total cost of ST. The cost per SFD gained is equal to the net costs for LEV treatment per person per year, divided by the net SFD gained. The cost per QALY gained in this study is equal to the net costs for LEV treatment per person per year, divided by the net QALYs gained.

### Sensitivity analyses

In order to test the robustness of the model and to evaluate the effect of uncertainty around model parameters on the ICER, we conducted extensive sensitivity analyses on all the variables in this study. To test the cost parameter estimates, costs for inpatient, outpatient and pharmacy and the acquisition price for LEV were both increased and decreased by 50%, the range that endorsed by the SAB. For parameters associated with transitions from or to certain doses of LEV, a full range (0-1) of two probabilities of better response (originally 0.5) and worse response (originally 0.8) were considered.

## Results

### Characteristics of patients for the trial in Korea^12^

Of a total of 100 Korean patients, 52 (52%) were male and 48 (48%) were female. Patients' mean age (±SD) was 35.3 (±11.7) years, while the mean age (±SD) at onset of epilepsy was 17.8 (±9.5) years. Eighty patients presented with at least one complex partial seizure, 28 patients with at least 1 secondarily generalized tonicclonic seizure, and 13 patients with at least 1 simple partial seizure during the baseline 3-month period.

In the history of previous AED treatment, 76 patients had taken two or more AEDs prior to entry to the study. The majority (80%) of patients entered the trial on two concomitant AEDs. The most frequently used concomitant AEDs were carbamazepine, valproic acid, lamotrigine, and topiramate.

### Cost-effectiveness analysis

#### Effectiveness Estimates

A patient treated with combination therapy (LEV+ST) had 340.7 SFDs per year, while a patient treated with ST had 322.4 SFDs per year. The incremental effectiveness is therefore 18.3 SFDs per patient per year (Table 2).

#### Cost Estimates

Over 1 year of treatment, the inpatient cost for combination therapy (LEV+ST) is only 64.5% of the inpatient cost for ST alone (US$ 1,184 vs. US$ 1,836 respectively). The additional cost of LEV for combination therapy is US$ 1,443. This shows that the acquisition cost of LEV is partially offset by its effectiveness. A patient on combination therapy (LEV+ST) costs US$ 3,178 per year, while a patient on ST alone costs US$ 2,380 per year. The incremental cost of treating patients with LEV add-on is therefore US$ 798 per patient per year (Table 3).

#### Cost-Effectiveness Ratio

The ICER (=ΔC/ΔE) is US$ 44 per SFD gained per patient for LEV add-on (Table 4).

#### Sensitivity Analyses

The results of the sensitivity analyses indicated the robustness of the model (Table 4). The range of the ICER for outpatient costs (±50%; 39-49) and pharmacy costs (±50%; 34-54) were relatively narrow, meaning that these two costs had less influence on the cost per SFD gained, equivalent to the ICER in the study. The cost ranges for inpatients (±50%; 17-70) and the price of LEV (±50%; 11-77) were wider. Although we made the conservative assumption that 100% of inpatient cost is expected when seizure frequency is zero (a state of P^better response^=0), the ICER was US$ 77 per SFD. Further, even when we assumed that patients with worse response needed 100% of inpatient costs (a state of P^worse response^=1), the ICER was US$ 45 per SFD (Table 4).

### Cost-utility analysis

#### Global Evaluation Scale and 31-Item Quality-of-Life in Epilepsy Inventory

The Korean clinical trial reported great improvement in global evaluation: improvement in 81% (marked, 41%; moderate, 16%: slight, 24%); no change in 16%; and worsening (slight) in 3%.^12^ No cases with moderate or marked worsening of the disease were reported.^12^

#### Quality Adjusted Life Years Gained and Net Cost

All patients in the combination therapy group gained 0.6522 QALYs per year, while those in the ST alone group gained 0.5801 QALYs per year (Table 2). The incremental utility was 0.072. As with the cost-effectiveness analysis, the incremental cost of treating patients with LEV add-on is US$ 798 per patient per year. Therefore, cost per QALY gained (=ΔC/ΔE) was calculated to be US$ 11,084 (Table 4).

#### Sensitivity Analyses

The results of sensitivity analyses supported the robustness of the model for cost-utility analysis (Table 4). The range of the cost per QALY gained for the outpatient costs (±50%; 9,811-12,358) and the pharmacy costs (±50%; 8,528-13,641) were relatively narrow, meaning that these two costs had less influence on the cost per QALY gained. However, the range for the inpatient cost (±50%; 4,423-17,745) and the price of LEV (±50%; 2,683-19,493) was wider (Table 4).

When we assumed that 100% of inpatient cost is expected even when seizure frequency is zero (a state of P^better response^=0), the cost per QALY gained was US$ 19, 523. Further, when we assumed that patients with worse response needed 100% of inpatient costs (a state of P^worse response^=1), the cost per QALY gained was US$ 11,544 (Table 4).

## Discussion

We found that treatment of refractory partial epileptic patients with LEV add-on therapy would be expected to increase the overall direct medical expenditures, but that the additional resources spent on extra drug costs would be partially offset by savings resulting from decreases in the utilization of other healthcare options (e.g., hospitalization). The model assumed the pathways followed by patients in actual clinical practice. The health outcomes considered were based on data derived from a clinical trial in Korea. All assumptions related to the information that was not available in the trial were clearly specified and were approved by the independent panel of experts (SAB). Moreover, the model proved to be flexible and can be readily adapted to different practice settings and different cost structure.

### Economic evaluation of antiepileptic drug addon therapy

The main health outcome of the analysis, i.e., SFDs, was improved by LEV with an incremental gain of 18.3 days per patient per year, thus resulting in a cost-effectiveness ratio of US$ 44 per SFD gained per patient per year. The ICER in this study looks grossly better than the Canadian report of CAN$ 80.7 per SFD gained.^19^ The superior ICER obtained here probably reflects different direct medical costs between Korea and Canada. Both models (Blais et al.^19^ and this study) share a common methodology using a decision-tree model for LEV add-on to ST for refractory epileptic patients and considering direct medical costs and SFDs gained as effectiveness. LeLorier et al.^23^ reported the potential benefit of seizure freedom (one additional seizure free patient per year) could be gained with a cost of £5,301 by adding LEV to standard treatment when considering the potential benefit of epilepsy surgery in the model. For other AEDs, Markowitz et al.^22^ found that lamotrigine (LTG) therapy cost an additional US$ 728 compared with the patients' current monotherapy only, discounted at 3% over 10 years. LTG therapy resulted in a gain 106 additional SFDs (undiscounted) and an ICER of US$ 6.9 per SFD gained. However, Markowitz et al.^22^ used the 10-year long-term model, but they did not discount outcome (i.e., SFD). Therefore, direct comparison of cost per SFD with a 1-year time horizon may not be justified. Schachter et al.^24^ estimated that adjunctive tiagabine (TGB), added to existing treatment of phenytoin, cost US$ 719 over 16 weeks including the cost of managing adverse events, compared with US$ 784 for adjunctive carbamazepine (CBZ). CBZ was more clinically efficacious (50% reduction in seizure frequency) but more detailed results were not provided. Within the baseline CBZ arm, add-on phenytoin (PHT) costs US$ 810 compared with US$ 958 for add-on TGB. Add-on PHT and add-on TGB had similar efficacy. Compared with current medication only, Messori et al.^25^ found that adjunctive LTG cost an additional US$ 1,612,370 for a cohort of 100 patients over the patients' lifetimes, gained 39 QALYs, and calculated an ICER of US$ 41,343 per QALY. When we consider both results of this study, previous economic evaluations of other AEDs, and the Gross Domestic Product of Korea (US$ 19,722/year/person, 2006), LEV is comparatively cost-effective against older AEDs.

### From disease-specific quality of life measurement to utilities

QALYs require a uni-dimensional measurement of QoL using both direct (i.e., standard gamble, time trade-off, person trade-off, visual analogue scale) and indirect utility assessments (i.e., EQ-5D, SF-6D, HUI). But, health-related QoL should be considered multidimensional, so it is often measured by disease-specific QoL scales. Therefore, analysis would be facilitated by converting disease-specific QoL measurement into utilities. Conversion algorithms were derived using 1 of 4 techniques: 1) transfer to utility regression, 2) response mapping, 3) effect size translation, and 4) revaluing outcome measures using preference-based scaling techniques.^26^ The resultant health state classification system consists of several dimensions (i.e., mobility, usual activity, visual acuity, pain, cognition, depression) with several levels (i.e., level 1=no problem, level 2=some problem, level 3=extreme problems), thus a generating huge number of health states. The valuing processes used in this study may be considered relatively new. Contrary to usual methods, levels were not categorized in this study, even though we used quality weights for 7 dimensions which were provided by original authors of QOLIE.^17^ Instead, subscale scores ranging from 0 to 100 were used to represent multidimensional levels of the health state of a patient. In this study, it was assumed that level is a continuous (linear), not categorical (stepwise) variable. Whether linear or stepwise, a higher score or higher level reflects more severe problems. A utility score was computed by summing up all utility portions of different dimensions in this study, which was adopted from the methodology for the EQ-5D Korean version.^27^

### Limitations

This study has some limitations. First, as with other cost-effectiveness studies, with regard to external validity, there is the problem of extrapolating clinical trial data (efficacy) to real world clinical practice (effectiveness). This problem was addressed through the sensitivity analyses. Second, because of the third-party payer perspective employed in this analysis, indirect costs were not considered in the model, even if they were estimated to represent a large proportion of the total costs associated with epilepsy. In order to gather such information, data derived from a long-term observation period must be used for this refractory population. Third, the Korean translation of the QOLIE-31 has not yet been validated, so all results of the cost-utility analysis should be considered in the light of data from relevant preference-based health economic studies. Fourth, we should use quality weights from other countries given that there are no prior studies that have been carried out in Korea. Special caution is needed when transferring these data to other contexts.

## Figures and Tables

**TABLE 1:**
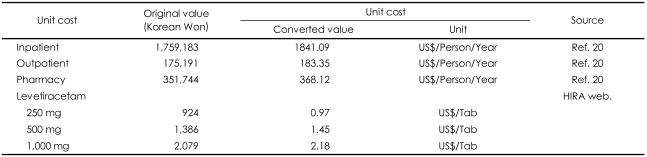
Unit costs for model input for the economic evaluation of levetiracetam add-on compared with standard therapy alone for the treatment of refractory epilepsy

HIRA: Health Insurance Review & Assessment Service (http://www.hira.or.kr). The average currency exchange rate in 2006 was 955.51 Korea Won per US$ 1 (http://www.keb.co.kr/IBS/welcome.jsp)

**TABLE 2:**
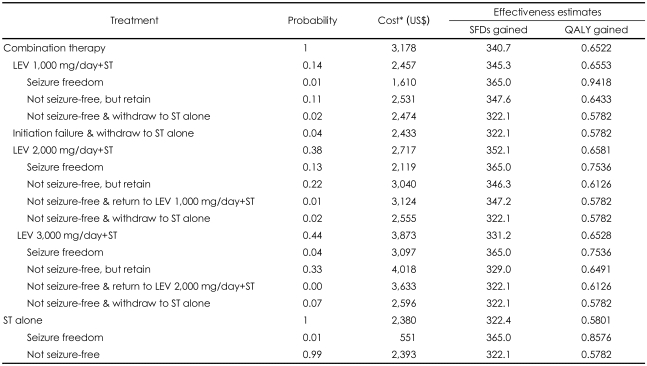
Probability, cost (US$/year/patient), SFDs and QALYs estimates used in the model

^*^Cost estimates for each state of combination therapy consisted of the sum of costs for inpatient, outpatient, and pharmacy plus drug acquisition cost for each dosage of LEV. Lower costs for each state of combination therapy (than sum of all four costs) were due to reduced health resource utilization after use of LEV. LEV: levetiracetam, SFD: seizure-free day, ST: standard therapy, QALY: quality-adjusted life year

**TABLE 3:**
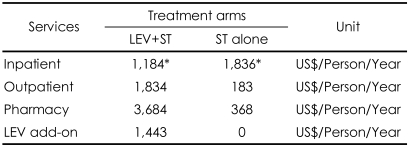
Cost comparison in patients with combination therapy (LEV+ST) versus ST alone of base case scenario

^*^These values are derived from the calculation multiplying unit cost for inpatient treatment with the probability that a person with refractory epilepsy will need hospital admission during 1 year. LEV: levetiracetam, ST: standard therapy

**TABLE 4:**
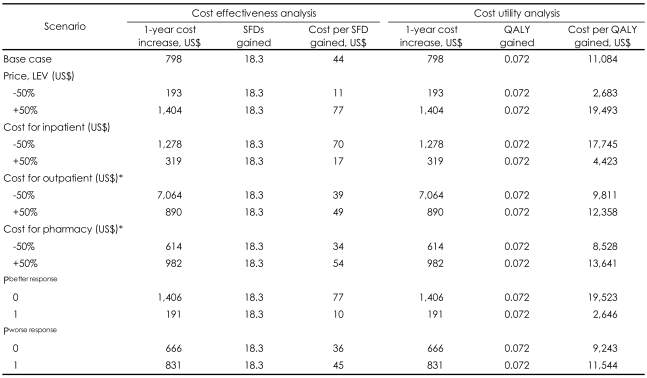
Incremental cost and health outcomes associated with the use of LEV. Base case and one-way sensitivity analysis

^*^These sensitivity analyses were conducted on the assumption that ST arm remained at base case scenario when combination therapy arm was being changed. LEV: levetiracetam, P^better response^: probability for better response in the group with better response (i.e., more than 50% reduction in seizure frequency)(0.5 at base case), P^worse response^: probability for worse response in the group with worse response (0.8 at base case), SFD: seizure-free day, ST: standard therapy, QALY: qualityadjusted life year
